# Severe Hypothermia in the Sunshine State

**DOI:** 10.7759/cureus.5088

**Published:** 2019-07-06

**Authors:** Amninder Singh, Tej G Stead, Rohan Mangal, Paul Banerjee, Latha Ganti

**Affiliations:** 1 Emergency Medicine, University of Central Florida / Healthcare Corporation of America Graduate Medical Education Consortium, Orlando, USA; 2 Emergency Medicine, Brown University, Providence, USA; 3 Emergency Medicine, John Hopkins University, Baltimore, USA; 4 Emergency Medicine, University of Central Florida, Orlando, USA; 5 Emergency Medicine, Envision Physician Services, Orlando, USA

**Keywords:** hypothermia, florida

## Abstract

The authors present a case of accidental hypothermia in a region with a warm climate. The article includes a review of the stages of hypothermia as well as the management of hypothermia. Clinicians need to be vigilant for this condition even in places with warm weather.

## Introduction

Primary accidental hypothermia is a drop in the core temperature to below 35°C. There are four stages of hypothermia. Mild hypothermia (Stage I) clinically presents with temperature ranging between 32°C and 35°C in a conscious and shivering patient. Moderate hypothermia (Stage II) is defined as temperature ranging between 28°C and 32°C. Patients with moderate hypothermia may have impaired consciousness. Severe hypothermia (Stage III) is distinguished by unconsciousness with the presence of vital signs and temperature from 24°C-28°C. Stage IV is defined as hypothermia with absent vital signs [1].

## Case presentation

A woman in her 70s with a past medical history of chronic obstructive pulmonary disease (COPD), coronary artery disease, hypertension, bipolar disorder, schizophrenia, and alcohol abuse was brought in by ambulance after she was found down. She was found unresponsive outside of her home early in the morning, and the ambient temperature was 12.8°C (55°F). The patient was bradycardic, with a heart rate in the 40s, blood glucose of 64 mg/dL, and blood pressure of 109/71 mmHg. She had a Glasgow Coma Scale (GCS) score of 3 with no gag reflex. The paramedics were unable to measure her temperature, as hers was too low to be read by their tympanic thermometer. The patient felt cool to touch. Upon arrival in the emergency department (ED), her heart rate was 36 beats per minute, blood pressure was 116/65 mmHg, and the patient was actively being ventilated by the paramedics with a bag valve mask. A temperature-sensing Foley catheter yielded a core temperature of 23.3°C (73.9°F). Her blood alcohol level was 90 mg/dL.

The patient was immediately intubated for airway protection. She was actively warmed with warm intravenous (IV) fluids, convection warming blankets, and an invasive femoral temperature management catheter. The patient had a history of being on beta blockers per her medical record. Glucagon was administered in case of an underlying beta-blocker overdose. The patient continued to be hypotensive and bradycardic in the ED, despite fluid resuscitation. She was started on a low-dose dopamine and dobutamine drip and admitted to the intensive care unit (ICU).

About 12 hours into her ICU stay, the temperature increased to 32°C (89.6° F). She was awake, remained intubated, and followed commands. She was still acidotic with a pH of 7.1. The following day, she was extubated. She was discharged home a few days later, neurologically intact, with a diagnosis of severe hypothermia, metabolic acidosis, and alcohol abuse.

## Discussion

**Figure 1:**
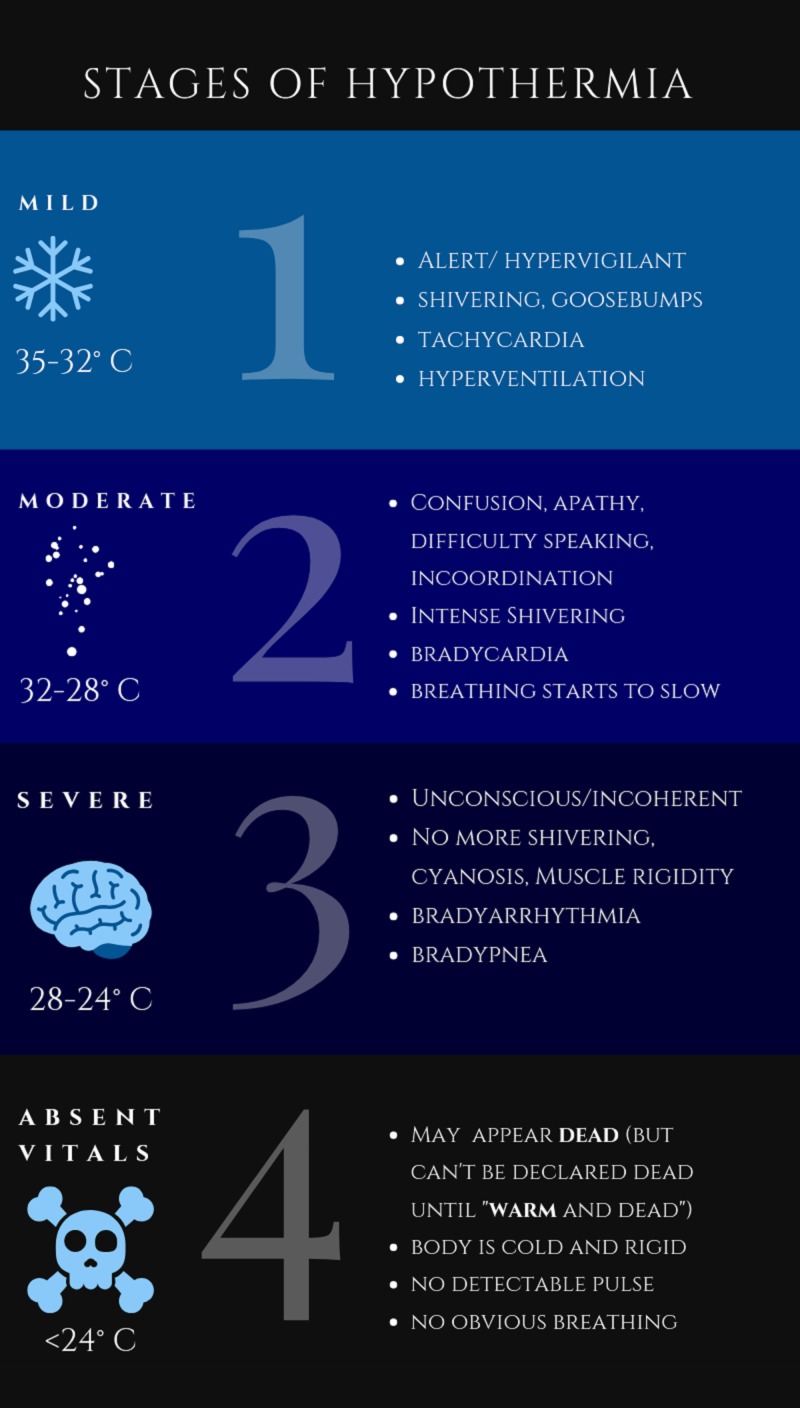
The four stages of hypothermia Graphic design by Karthik D. Stead

The management of hypothermia initially begins with paying close attention to the airway, breathing, circulation, and re-warming. It can be difficult to assess for signs of life, especially in the later stages of hypothermia (Figure 1). Peripheral pulses are usually diminished due to vasoconstriction. It is important to check central pulses for signs of life, and for at least one minute prior to starting cardiopulmonary resuscitation, as hypothermic patients can be severely bradycardic.

Rewarming should be started with passive and active core re-warming. Care must be taken not to warm the extremities initially, to prevent core temperature afterdrop, which occurs when the cold and acidotic blood present in the vasoconstricted extremity returns to the core circulation [2-3]. In our patient, we used an invasive femoral vein catheter to perform internal core re-warming. The femoral catheter circulated warm water until a target temperature of 34°C was achieved, with the goal of increasing the core temperature by 2°C/hour. In hypothermic patients, the myocardium is very sensitive; hence, the placement of a central venous catheter in the internal jugular or subclavian vein is not preferred. The guidewire can irritate the myocardium and cause lethal dysrhythmias such as ventricular fibrillation or ventricular tachycardia. Also, care must be taken to avoid rough or unnecessary movement so as not to precipitate dysrhythmias [3]. The patient should also be transported horizontally as changes in position can affect venous return, which can lead to cardiovascular collapse [3].

Our patient remained hypotensive despite fluid resuscitation. We used dopamine and dobutamine as our first line of pressors. We did not want to use norepinephrine, as hypothermic patients are peripherally vasoconstricted. We did not want to risk limb ischemia with norepinephrine. There is limited literature on the choice of pressor in hypothermia. In swine studies with induced hypothermia, low-dose dopamine and dobutamine have been used to increase cardiac output, with no risk of inducing arrhythmias up to a max. dose of 30 micrograms/kg/min [4].

## Conclusions

We describe a case of an elderly female with severe hypothermia of 23°C. The patient had completely neurologically intact survival to discharge. In accidental hypothermia, care begins with avoiding aggressive movement, keeping the patient horizontal, passive, with active core rewarming and close attention to the patient’s airway, breathing, and circulation. Vasoactive pressors, such as dopamine and dobutamine, can be used in the persistently hypotensive patient with severe hypothermia.
